# Progress needed on climate and health goals, Timor-Leste

**DOI:** 10.2471/BLT.25.293603

**Published:** 2025-05-01

**Authors:** José Ramos-Horta, Unni Karunakara, Remco van de Pas

**Affiliations:** a Presidéncia da República, Dili, Timor-Leste.; b Global Health Justice Partnership, Yale Law School, New Haven, United States of America.; c United Nations University International Institute for Global Health, Hospital Canselor Tuanku Muhriz UKM, Jalan Yaacob Latif, Bandar Tun Razak, Cheras, 56000 Kuala Lumpur, Malaysia.

Timor-Leste is highly vulnerable to the climate crisis. Rising sea levels, flooding, landslides, droughts and biodiversity loss are already threatening lives, livelihoods and property.^1^ Concurrently, the country is recovering from 25 years of conflict and faces considerable health challenges, including high prevalence of tuberculosis and malnutrition, and limited access to quality health care.^2^ In Timor-Leste, climate change is not only an environmental issue but a health crisis that requires action.^3^

In recent years, the interconnectedness of climate and health has gained prominence on Timor-Leste’s national agenda. Furthermore, Timor-Leste’s potential membership in the Association of Southeast Asian Nations (ASEAN) and its leadership within Small Island Developing States and least developed countries provide opportunities to leverage international partnerships for transformative change.^4^^,^^5^

The climate crisis directly affects the health of Timor-Leste’s population, which is expected to increase by more than one third by 2050.^2^ The demographic and climatic pressures will disrupt agriculture, the country’s primary livelihood source. Environmental stressors will worsen food insecurity and malnutrition, increasing the incidence of communicable and noncommunicable diseases. For instance, floods can cause water contamination and diarrhoea, while warmer temperatures facilitate the breeding of disease-carrying mosquitoes.^2^ Addressing these challenges requires building climate resilience and integrating climate adaptation into the national health system.

Timor-Leste’s economy is heavily reliant on its sovereign Petroleum Fund, which faces depletion by 2035.^6^ However, excessive dependence on oil has put the country’s economy at risk,^7^ necessitating economic diversification. A just transition to renewable energy is essential for long-term sustainability, reducing reliance on petroleum while mitigating climate impacts. Successfully managing this transition without compromising investments in vital sectors such as higher education, public health care and maritime biodiversity protection will be key to unlocking the full potential of Timor-Leste’s demographic dividend. To maintain fiscal space, the country must leverage remittances from its skilled diaspora and harness opportunities through fair trade, responsible eco-tourism and development assistance. Moreover, Timor-Leste has the potential to serve as a model for sustainable development – one that prioritizes social and ecological well-being, gender equity and human security, whilst avoiding the pitfalls of unchecked growth.^8^ In this way, the country can align with the example set by other island nations such as Iceland and New Zealand.^9^ This approach supports Timor-Leste’s stance, seeking international recognition that countries have a common but differentiated responsibility in addressing climate change.^10^

To tackle climate and health challenges, the government of Timor-Leste must integrate climate mitigation, adaptation, and loss and damage strategies with robust health policies in five key areas. 

First, strengthening climate-resilient health systems. Doing so includes enhancing early warning systems for climate-related health risks, boosting health-care facilities’ capacity to respond to natural disasters and ensuring the availability of medical supplies and trained personnel.

Second, climate-smart agriculture and health interventions. Agriculture and health are deeply interconnected in Timor-Leste. The government should invest in climate-smart agricultural practices that promote food sovereignty and reduce malnutrition. Investments could involve developing irrigation systems and sustainable farming techniques that are both climate-resilient and health-promoting, such as increasing nutrient-dense crops and minimizing pesticide use.

Third, access to quality health care. Timor-Leste needs sustainable investments in health services from the primary to tertiary levels. Collaboration with Cuba, Lusophone nations, ASEAN and others will be vital for achieving universal health coverage.

Fourth, strengthening regional and international cooperation. Continued engagement with ASEAN, small island developing states and international organizations will be crucial for accessing research, technology transfers and funding. 

Fifth, public awareness and education. Raising awareness about the link between climate change and health is critical. 

Timor-Leste faces complex challenges at the intersection of climate change and health. By aligning transformative climate action with health policies, its government can set an example for other small island developing states facing similar threats.
